# To deny, to justify, or to apologize: Do social accounts influence stress levels in the aftermath of psychological contract breach?

**DOI:** 10.1186/s40359-020-00505-2

**Published:** 2021-01-06

**Authors:** Safâa Achnak, Arjen Schippers, Tim Vantilborgh

**Affiliations:** 1 Work and Organizational Psychology (WOPs), Vrije Universiteit Brussel, Pleinlaan 2, 1050 Brussels, Belgium; 2 Experimental Psychology, Vrije Universiteit Brussel, Pleinlaan 2, 1050 Brussels, Belgium

**Keywords:** Psychological contract breach, Stress, Heart rate, Social accounts, Trajectories

## Abstract

**Background:**

Workplace stress carries considerable costs for the employees’ wellbeing and for the organization’s performance. Recent studies demonstrate that perceptions of psychological contract breach are a source of stress for employees. That is, when employees notice that their employer does not fulfil certain obligations, they will perceive that certain resources are threatened or lost, which in turn translates into increased stress. In this study, we zoom in on how stress unfolds in the aftermath of breach, dependent on the organization’s reaction to the breach. More specifically, we examined the influence of different types of social accounts (i.e., denial, apology, blaming and exonerating justification) on individuals’ stress resolution process using physiological (i.e., heart rate) and psychological (self-report) data.

**Method:**

We used an experimental design in which we manipulated psychological contract breach and social account type. To test our hypotheses, we performed two sets of functional Principal Component Analyses: first to examine the effects of breach and second to examine the effects of social accounts.

**Results:**

Our results indicate that breach elicits a physiological stress reaction, reflected in a short-lived increase in heart rate. However, no increase in the self-reported stress measure was found. Further, we did not find a significant effect of social accounts on the psychological and physiological recovery process.

**Conclusions:**

The current research allows us to demonstrate that psychological contract breach will trigger a short-lived increase in heart rate. Further research is needed to better understand unfolding trajectories of physiological reactions to contract breach and the effect of social accounts as organizational recovery efforts.

## Background

The psychological contract (PC) has been defined as a continuous exchange of a set of reciprocal obligations between an employee and an employer [52, 55] shaping the current and future state of the employee-employer exchange relationship [15]. The PC is considered a critical construct in organizational behaviour literature because employees who perceive that their employer has failed to fulfil one or more obligations may perceive a PC breach (PCB), which is often associated with feelings of violation (i.e., a mixture of negative emotions such as anger and frustration; [48]. Although substantial empirical progress (for a meta-analysis see [74] has been made in understanding the relationship between PCB, violation feelings, and employee attitudes (e.g., reduced job satisfaction, organizational commitment) and behaviors (reduced performance and increased turnover; e.g., [14, 30, 50], little attention has been given to the role of time in understanding this chain of events. That is, PC research has been predominantly contemporaneous and, in doing so, has overlooked the temporal context in which perceptions of PCB are formed, and in which employee reactions unfold over time (see [1]. Furthermore, although prior research has mainly focused on the relationship between PCB and employee attitudes or behaviors, far fewer studies have investigated its influence on employee wellbeing in terms of stress. Previous research has demonstrated that PCB triggers stress reactions [2] because employees may consider a PCB as a (potential) loss of valued resources (Conservation of Resources Theory; [26], which is considered a stressful event [49]. Repeated stress can have harmful effects for the individual (e.g., poor mental health [22]) and the organization (e.g., absenteeism; [24]. Hence, a better understanding of the relationship between PCB and stress is imperative to avoid the detrimental consequences resulting from stress reactions associated with PCB.

Therefore, moving beyond the immediate stress reactions associated with perceptions of PCB, we aim to examine how post-PCB stress levels unfold over time during the course of an experimental study. Examining the unfolding nature of stress trajectories in the aftermath of PCB is important as perceptions of PCB may trigger an immediate increase in stress levels [2], it is unlikely that stress will remain at this elevated level perpetually. Indeed, the recent Post-Violation Model (PVM; [66] suggests that responses to PCB and violation fluctuate over time. More specifically, the PVM states that violation victims use self-regulation processes in an attempt to deal with PCB through resolution efforts. Eventually, four possible PC outcomes can result from such efforts, ranging from highly functional (i.e., PC thriving) to highly dysfunctional PCs (i.e., PC dissolution). Building on this premise, we argue that some people may recover from a PCB and return to pre-PCB stress levels, whereas others may become trapped in a prolonged state of stress.

Next, while most PC research is primarily focused on how employees react to perceptions of PCB and how these reactions negatively impact employees and organizations, far fewer studies have focused on how organizational actions might influence how employees react to perceptions of PCB, and how they may overcome the stress that is otherwise associated with these perceptions. In this paper, we build on the organizational fairness (e.g., [4, 5] and trust repair literature (e.g., [36, 62]) to explore the success of various social accounts as a mechanism to overcome the stress reactions associated with PCB. For example, trust recovery efforts are more efficient when organizations offer monetary compensation and sincere apologies [17, 59]. Employees who perceive that their organization engages in recovery efforts are more confident regarding the resolution process and will tend to view their organization as trustworthy [59]. The primary purpose of the current investigation is to gain a better understanding of how unfolding stress reactions following PCB can be influenced by organizational interventions. We experimentally examined how different types of organizational social accounts can alter the stress resolution/recovery process after perceiving a PCB. In doing so, we do not only provide an empirical test of resolution attempts in the aftermath of PCB, but also provide practitioners and policy makers with valuable information about which organizational interventions they should develop and implement to reach more successful resolution outcomes.

Further, to gain a broad understanding of stress responses following PCB, we use a combination of both physiological and self-reported measures. Most existing PC studies assessing emotional and stress reactions to PCB employed retrospective self-reported measures. However, memories of emotions are subject to systematic biases. Indeed, it appears that individuals are often influenced by their current feelings about appraisals of past events when reporting about their previous emotional reactions to those events [41]. Therefore, using physiological measures leads to a more objective and unbiased evaluation of reactions to PCB. Moreover, physiological responses to stress are important determinants of health as a stressful stimulus results in the activation of several physiological pathways including the autonomic nervous system (ANS). A considerable body of research has linked the function this system with the pathogenesis of physical, behavioral, and mental health symptoms (e.g., [19, 38, 58, 73]. The most commonly assessed indices of ANS activation are based on electrodermal (i.e., skin conductance level) or cardiovascular (e.g., heart rate, blood pressure, heart rate variability) responses. Previous research [25, 33, 39] has indicated that a psychosocial stressor involving the ego and a social-evaluative judgement by others (i.e., a stressor similar to the one induced in the present experiment) stimulates the sympathetic nervous system (i.e., a branch of the ANS) as assessed by heart rate (HR). We therefore assessed in addition to psychological stress, participants’ physiological reactions through their HR.

### Psychological and physiological stress responses to psychological contract breach

As noted previously, in their conceptual model, Morrison and Robinson [48] distinguished between perceptions of PCB and violation feelings when proposing that violation feelings would mediate the relationship between PC breach and employee attitudinal and behavioral outcomes. Meta-analytic research [74] has indeed demonstrated that feelings of violation are a key mediating mechanism between PCB and employee attitudes and behaviors. More recently, Achnak et al. [2] demonstrated that PCB does not only evoke negative emotions but also triggers stress reactions. This positive association between perceptions of PCB and stress can be explained by drawing upon Conservation of Resources Theory (COR, [27]). According to COR theory, employees have a need and desire to maintain valuable resources. Resources are “*those objects, personal characteristics, conditions, or energies that are valued by the individual or that serve as means for attainment of these objects, personal characteristics, conditions, or energies”* ([27], p. 516). When employees experience a loss, potential loss, or failure to gain resources, stress reactions are evoked [27, 32]. In line with recent research [2, 49], employees consider the PC as an agreement to exchange resources and may thus experience a PCB as a (potential) loss or a failure to gain resources. For example, employees may legitimately believe that their organization owes them job security. If the organization fails to fulfill this obligation, employees’ resources and possibly their capacity to protect their current personal lifestyle may be threatened [49], leading to the development of stress reactions. In contrast, when the organization does fulfill its obligations, employees are capable of maintaining and/or acquiring desired resources, which will prevent them to experience such reactions [26]. While previous research has demonstrated the positive linkage between PCB and psychological stress [2, 49], this study aims to go a step further by establishing a causal effect of PCBs on both subjective and physiological stress responses, hypothesizing the following:

- **Hypothesis 1a**: PCB cause increased physiological activity compared to PC fulfillment.
- **Hypothesis 1b**: PCB cause increased subjective stress compared to PC fulfillment.

### How can social accounts influence stress resolution?

According to the PVM, employees will reach a state of resolution when the perceived PC discrepancy and the negative consequences that arise from it are eliminated. Whether the resolution process will be successful or not depends among others on how the organization responds to the PCB [66]. Following the trust repair literature, this organizational response, in form of a social account, will influence employees’ attitudinal, affective and behavioral reactions to a negative work outcome [21, 37]. Social accounts can be described as attempts to shape employees’ perceptions following a negative event [64]. According to Bies [6] organizations can respond to perceptions of PCB by means of social accounts that aim to bridge the gap between what employees initially expected and what they actually perceived. This could be achieved by for example providing a suitable and honest explanation for a negative (work) event because said explanation tends to lead to the experience of higher fairness perceptions and lower resentment, compared to when that event remains unexplained or inadequately explained [5, 61]. Analogous to Tomlinson and Mayer [64] research on trust recovery, the present study aims to examine which type of social account is likely to be most effective for the stress resolution process in the aftermath of a PCB. Previous research has demonstrated that accounts that refer to the cause of the negative event (i.e., PCB) as being (1) not controlled by the organization, (2) external to the organization, and (3) unstable over time are more likely to exonerate the organization of blame [70, 71]. In addition, organizational justice researchers [6, 13, 72] have provided valuable information regarding different typologies of social accounts that individuals use to improve their damaged reputation. In the present study we focus on three distinct types of social accounts also used in Tomlinson and Mayer [64] research: denial, justification, and apology.

First, the organization can deny the existence of the PCB. In doing so, the organization affirms that they are not accountable for this negative event and therefore should not be held responsible for it [6, 13]. However, in doing so, employees are likely to develop, and sustain, negative affective reactions [7, 21] Specifically, employees may end up in an uncertain position regarding which current and future resources they may or may not expect from their organization. They have no guarantee that the negative outcome will not reoccur and might come to believe that their organization deliberately failed to fulfil its obligations towards them. This uncertainty about the preservation and gain of resources will inherently lead employees to experience continuous stress [26]. Hence, we hypothesize the following:

**Hypothesis 2**: The stress resolution process following a denial will be less successful.

Alternatively, the organization can justify the PCB, and in doing so the organization tries to minimize its responsibility for the PCB [6]. The organization will try to debilitate its accountability by claiming attenuating circumstances [13]. In contrast to a denial, the organization acknowledges a part in the failure but states that it is not entirely responsible for it [60]. By providing a justification, the aim is to locate the cause of the PCB to factors that are less central to the organization [62]. However, not all types of justification are created equally, and different types of justifications can be identified. A justification indicating that the PCB’s cause is beyond the organization’s control and is unstable over time will increase the likelihood that the organization is exonerated of its blame [71]. Hence, this type of exonerating justification will signal that the (potential) loss of resources or failure to gain resources is an exception and that the situation will eventually be solved, which in turn will positively impact the stress resolution process. For example, suppose an employee expects his/her organization to allow him/her to have more flexible hours since (s)he recently became a parent but the organization fails to provide the employee with more flexibility. This PCB may represent a loss of valued resources (i.e., flexibility) and trigger stress reactions [2]. The organization can account for this negative work event by providing an exonerating justification and stating that the PCB is caused by a temporary high workload due to a sudden influx of extra customers. This type of justification minimizes the organization’s responsibility. In contrast, justifications that amplify the organization’s responsibility and controllability indicate that the (potential) loss of resources or failure to gain resources might endure. For example, the lack of flexibility provided by the organization can be justified by the absence of a qualified co-worker that can take over the job. This cause of the PCB is not entirely out of the organization’s scope of responsibility and controllability. The stress reaction to PCB is then less likely to be resolved. Therefore, we hypothesize that:

- **Hypothesis 3**: The stress resolution process will be more successful when the organization provides an exonerating justification.
  - **Hypothesis 4**: The stress resolution process will be less successful when the organization provides a blaming justification.

Finally, instead of using a justification, the organization may offer an apology in response to a PCB in an attempt to reframe the employee’s judgement after a negative work event [6, 13]. By apologizing, the organization admits its responsibility and expresses remorse for the inflicted harm [63]. Put differently, the dispositionally “well-intentioned” organization atypically “failed” to fulfill its obligations but will not reiterate this behavior [64]. Several studies support the idea that offering apologies after a negative outcome leads to more positive affective reactions [64, 65]. Like exonerating justifications, apologies communicate that there is no enduring damage resulting from the PCB. Hence, stress resulting from the perceived (potential) loss of or failure to gain resources is more likely to be resolved. Therefore, we hypothesize that:

- **Hypothesis 5**: The stress resolution process will be more successful when the organization provides an apology.

**Fig. 1:**
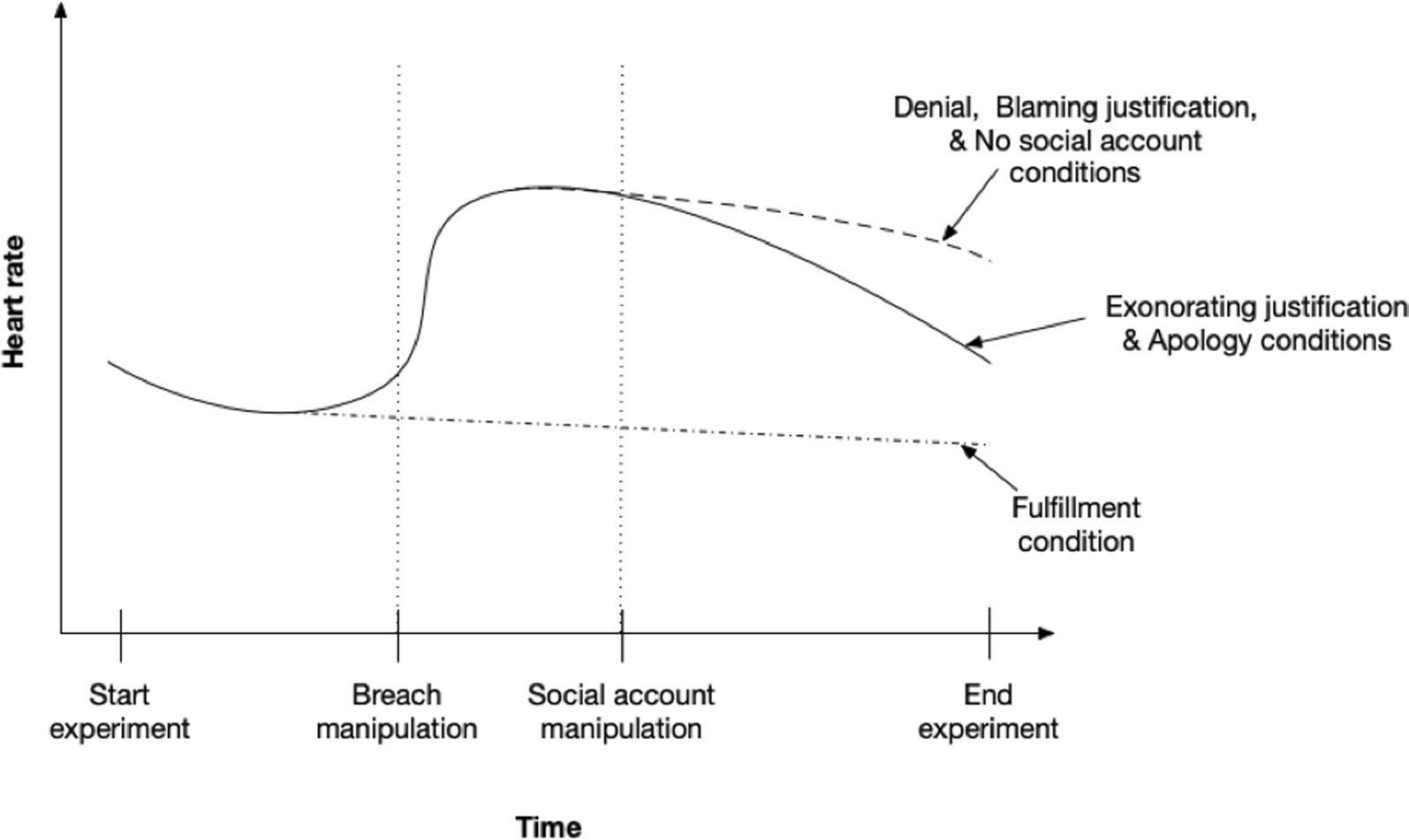
Hypothesized HR trajectories during PCB induction (H1a) and after different types of social account delivery (H2-5)

To visualize the proposed trajectories of stress resolution, we plotted the distinct hypothesized HR trajectories depending on the type of social account delivery (see Fig. 1).

## Methods

### Ethics approval

This experiment was approved by the human sciences ethics committee (ECHW2015-16) of the first author’s university. All participants signed a written informed consent prior to participation.

### Participants

We conducted this experiment on a sample of 105 undergraduate psychology students from a Belgian university, who participated in return for course credit. An a-priori power analysis suggested that this sample size would be sufficient to detect medium-to-large effects with 80% power. The majority of the sample was female (86.67%), the average age was 18.87 years (*SD* = 1.79), and 20.95% of the participants had work experience. Participants in the fulfilment condition were part of a second, different, experiment that used the exact same procedure, but in which no psychological contract breach was induced and, consequently, no social account was offered for that breach. There were no significant differences between the participants in the breach conditions and participants in the fulfilment condition in terms of gender (χ^2^(1) = 0.01, *p* = 0.93, *d* = 0.03), age (*t*(20.48) = 1.25, *p* = 0.23, *d* = 0.25), or work experience (χ^2^(3) = 2.28, *p* = 0.52, *d* = 0.16).

### Procedure

In the present experiment we attempted to create a PC between the experimenter and the participant. Before starting the experiment, we asked participants to complete a battery of questionnaires to assess whether they had recently consumed alcohol or drugs, or had any medical condition that may have biased the results (e.g., a heart condition). None of the participants met these exclusion criteria. In a next step, we used a cover story in which we informed students (initially participating in a psychological study in return for course credits) that they are participating in an experiment examining the effect of emotions on problem-solving abilities. We explained that in order to do so, they are asked to solve mathematical tasks (i.e., participants’ obligation) in exchange for payment according to their performance (i.e., experimenter’s obligations). This explanation established the core of the PC given that such a contract is defined as being the perceived agreement derived from promise-based obligations between two social parties [54].

Next, we instructed participants to perform a computer task, programmed in E-prime, which would be assessed by the experimenter who was located in another room and supposedly monitored their responses. The computer task consisted of a matrices task (24 matrices in total). Each matrix comprised a set of 12 three-digit numbers (see example in Table 1). Participants had 38 s to find the two cells that sum to ten. We computed these times based on the time sixteen college students needed to complete the 4 × 3 matrix tasks. We told participants that for each completed task, the experimenter would award them tokens based on both the accuracy and the speed of their responses compared to a fictitious norm group. After the completion of each task, we showed participants a message that displayed the amount of tokens they would receive from the experimenter. In reality, the amount of tokens was randomly determined a priori by the experimenters, and was not based on the actual performance of the participants. We made sure that the amount of awarded tokens was the same in each condition and for each participant. The participants were informed that each token was worth 0.10€ and that they would be paid for their performance at the end of the experiment depending on the total amount of collected tokens. This design is similar to real work life experiences such that employees receive a promotion, a pay raise or a bonus relative to their performance, and against which they evaluate their PC. Moreover, consistent with previous experimental research [40, 47], we chose for pay as a general resource type that satisfies other needs.

**Table 1:** Example of matrix with 12 three-digit numbers

3.47	2.70	4.11
2.36	8.89	6.63
1.32	9.84	4.90
1.11	1.65	7.40

Participants were randomly assigned to one of six conditions based on whether they experienced a psychological contract breach or not, and on the social account they were given after experiencing a breach (see “Appendix 2”): fulfilment condition (*n* = 19), breach—denial condition (*n* = 18), breach—exonerating justification condition (*n* = 19), breach—blaming justification condition (*n* = 16), breach—apology condition (*n* = 17) and breach—no social account condition (*n* = 16). Throughout the experiment, participants were connected to a NeXus 10-MKII recording device and pre-gelled Ag/AgCl electrodes to assess heart rate (HR). Before starting the actual experiment, participants were informed that they would complete four practice trials to accustom to the unfamiliar setting and to ask additional questions if needed. This practice block was followed by six experimental blocks, each comprising four matrices. Participants were not informed about the total number of experimental blocks. After each block, the experimenter communicated the total amount of tokens participants had gained, as well as the corresponding amount of money they had earned. Halfway the experiment (i.e., after three experimental blocks), the experimenter induced a PCB by announcing that participants would not be paid for their performance anymore. Nevertheless, they were expected to continue the experiment until the end. After completing an additional block, participants were asked whether the experimental leader fulfilled their obligations towards them, after which participants in the breach conditions were given one of four social account or no social account at all. Once the six blocks were completed, participants were instructed to fill out a second questionnaire assessing their emotions. Finally, participants were detached from the sensors and debriefed. Each participant received 10€ for participation.

### Measures

All surveys were provided in Dutch. We used a translation and back-translation process after which inconsistencies were discussed and resolved. All data were fully anonymized prior to analyses.

*General questionnaire measures*. We used a general questionnaire to collect demographic information on participants’ age (in years), gender (female or male), and professional background (current work status). Additionally, we collected information regarding the participants’ medical condition and substance use (medication and health complaints, caffeine, nicotine, soft drugs, and hard drugs) to check for exclusion criteria. We furthermore assessed other variables (i.e., Emotions [69], Rumination [67], Behavioral Inhibition and Activation System [11], and Equity Sensitivity [56]) that were however not used in the analyses of this study.

*Psychological contract breach.* We assessed perceptions of psychological contract breach using an adapted version of the two-item scale from Rousseau [52, 55]. The two items were: “Overall, the experimental leader fulfilled his commitments to me.”, and “In general, the experimental leader lived up to his promises to me.”. Participants were asked to indicate their response on a 5-point Likert scale ranging from (1) “Totally disagree” to (5) “Totally agree”. Reliability was adequate as the correlation between both items was *r* = 0.73 (*p* < 0.001). Items were reverse-scored so that high scores on this scale reflected strong perceptions of breach.

*Feelings of violation.* We measured feelings of violation, using an adapted version of Robinson and Morisson’s [51] four-item scale. An example item is “I feel a great deal of anger toward the experimental leader”. Participants were asked to indicate their response on a 5-point Likert scale ranging from (1) “Totally disagree” to (5) “Totally agree” (α = 0.88). High scores on this scale reflect strong feelings of violation. In line with the literature [74], we found a strong positive correlation between perceptions of breach and feelings of violation (*r*(102) = 0.64, *p* < 0.001).

*Physiological stress indicator.* Consistent with previous stress research (e.g., [18, 31, 45] physiological response to stress was measured through participants’HR. HR is one of the most commonly used indices of activation of the autonomic nervous system and has been shown to be an indicator of arousal [43]. We extracted HR by recording an electrocardiogram at 256 Hz using a NeXus 10-MKII recording device and pre-gelled Ag/AgCl electrodes.

*Subjective stress ratings.* In addition to physiological measures, we also assessed subjective stress ratings using a self-developed single-item scale, where participants had to indicate their current level of stress on a nine-point Likert scale, ranging from 1 (“Totally not stressed”) to 9 (“Extremely stressed”). This item was presented at the end of each block, yielding six observations for each participant over the course of the experiment.

### Analysis

**Fig. 2:**
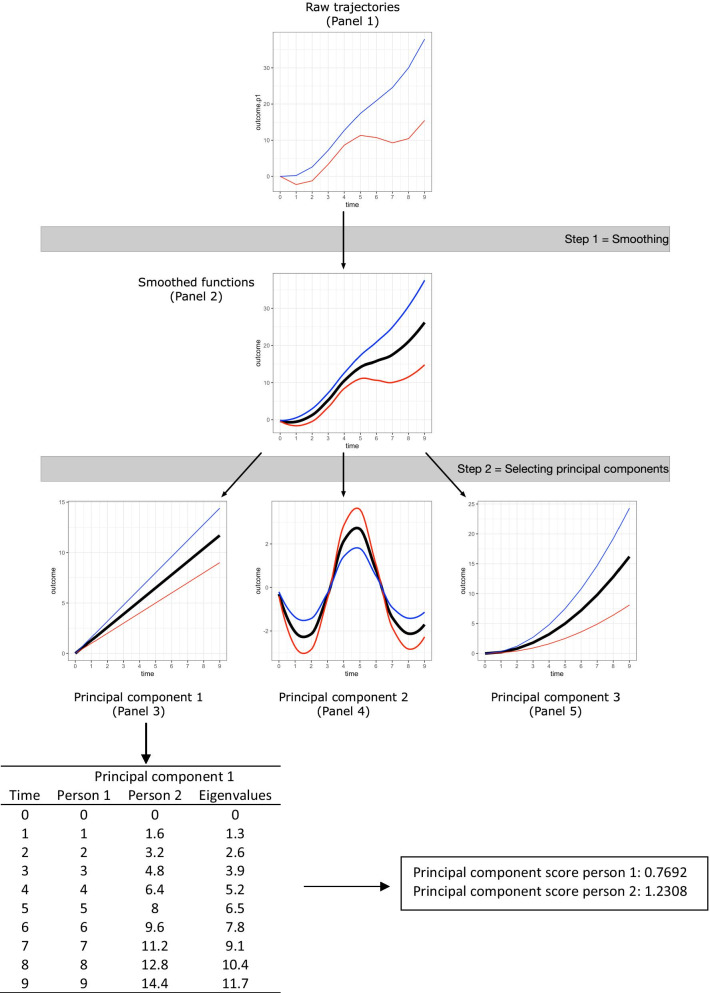
Illustration of functional principal component analysis steps. Black curves represent average trajectory (eigenvalues in panels 3–5), red curves represent trajectory of person 1, blue curves represent trajectory of person 2

We started by analyzing the HR data using functional Principal Component Analysis (fPCA), which belongs to the family of functional data analysis (FDA) techniques [16]. FDA is a relatively new methodology within the Human Resource Management and Organizational Behavior domains, but has several advantages when studying complex, dynamic phenomena [16, 28]. Primarily, FDA is ideally suited to analyse highly nonlinear and heterogeneous longitudinal data and can handle large data sets [16]. For example, the complex nonlinearity of HR data and the sheer amount of HR observations for each individual in our experiment (e.g., > 14,000 h observations for participant 1 following the social account manipulation) make it difficult to resort to more traditional approaches to analyze longitudinal data, such as latent growth models or random coefficient models [28]. FDA is able to handle such data by using a two-step process, which is illustrated in Fig. 2. For the purpose of this illustration, imagine that Panel 1 of Figure displays the trajectories of a variable over time for two individuals (person 1 = red trajectory; person 2 = blue trajectory). The goal of fPCA is to identify common modes of variation in these trajectories (i.e., principal components) and to determine how each participant scores on these principal components. In the first step, FDA generates a smooth continuous curve from the discrete observations of a variable (e.g., HR) for each individual (see Panel 2 of Fig. 2). Put differently, the raw observations are replaced by a curve or function for each individual, which then become the unit of analysis in the next step.

In the second step, fPCA identifies common modes of variation underlying these functions, called principal components. Essentially, fPCA shares the same underlying principles as ordinary PCA, namely identifying common factors, except that the unit of analysis are the curves extracted in step one [16]. In Fig. 2, the curves are decomposed in three principal components: the first capturing linear change over time (Panel 3), the second identifying sinusoidal change over time (Panel 4), and the third reflecting exponential change over time (Panel 5). Each principal component is described by eigenvalues, which capture the average trajectory of that principal component over time. The number of principal components that are selected is based on their explanatory power. We rely on the common rule of thumb that the selected number of principal components need to jointly explain 90% or more of the variance [29]. Each individual is assigned a principal component score on each of the extracted principal components. Continuing our example, the first principal component could capture linear change over time, but the individual scores on this principal component can inform the researcher that some individuals (e.g., Person 2; blue trajectory in Panel 3) experience a steeper linear increase than others (e.g., Person 1; red trajectory in Panel 3). Multiplying an individual’s principal component score with the eigenvalues of that principal component allows one to reconstruct that individual’s unique trajectory for the specific principal component (e.g., multiplying the eigenvalues of the first principal component with the principal component score of person 1 in Fig. 2, results in the linear change scores of person 1 over time for the first principal component). We subsequently used these individual principal component scores on each of the extracted principal components as dependent variables in a MANOVA, with the conditions as the independent variable. To analyze the subjective stress data, we relied on repeated-measures ANOVA. All analyses were performed in R, using the *fdapace* and the *afex* packages.

Prior to the analysis, we transformed the HR data into within-person z-scores [8]. In addition, each participants’ time variable was rescaled from 0 to 1 so that HR trajectories could be compared. The reason for this is that speed of responses given to the matrix tasks differed both within- and between-participants, meaning that the duration of each trial differed between participants. fPCA requires that all HR functions are measured on a common grid of design timepoints within a common interval *I* = [a, b]. Rescaling each participants’ time variable to an interval *I* = [0, 1] was therefore required.

The datafiles and the R scripts used to analyze the data can be downloaded from https://osf.io/qj86m/?view_only=8af8ef16ee3340a48b1ccb217bafd5e2.

## Results

### Manipulation checks

First, we assessed if we successfully induced a PCB in the breach conditions, by comparing scores on the PCB and feelings of violation measures between the breach conditions (*n* = 85, one participant did not provide ratings on the breach and violation measures and was excluded from this analysis) and the fulfilment condition (*n* = 19). We tested this with a MANOVA. When testing the assumptions for MANOVA, the Shapiro–Wilk test indicated that the residuals for the breach (*W* = 0.92, *p* < 0.001) and the violation (*W* = 0.84, *p* < 0.001) variables were not normally distributed. The Bartlett test showed that the assumption of homoscedasticity was also not met for the breach (*K*^*2*^ = 7.34*, df* = 1, *p* = 0.01) and violation (*K*^*2*^ = 39.44*, df* = 1, *p* < 0.001) variables. We therefore used a rank-based non-parametric MANOVA with 10,000 bootstraps. Results showed that there was a significant difference between the breach and the fulfillment conditions (*Test statistic* = 4.87, *p* < 0.001), with the breach condition scoring higher than the fulfilment condition on perceptions of psychological contract breach (*Unweighted treatment effects*: Breach = 0.61, Fulfillment = 0.39) and on feelings of violation (*Unweighted treatment effects*: Breach = 0.61, Fulfillment = 0.39). These results suggest that our manipulation of breach was successful.

Second, we checked if participants in the experimental sample were aware of the social account that was offered. This was done by asking participants during the debriefing to indicate if the experimenter offered an explanation for any broken obligations. None of the participants failed this manipulation check. In other words, all participants indicated they had received a social account when it was indeed the case.

### Functional data analysis

**Fig. 3:**
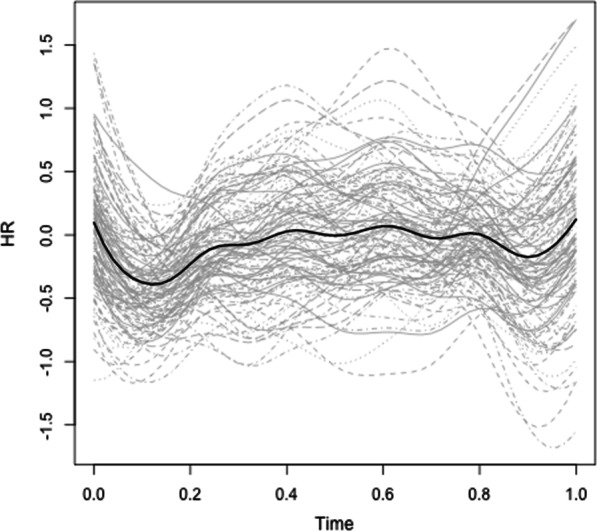
Individual (in grey) and average (in black) HR trajectories during and following the breach inducement

**Fig. 4:**
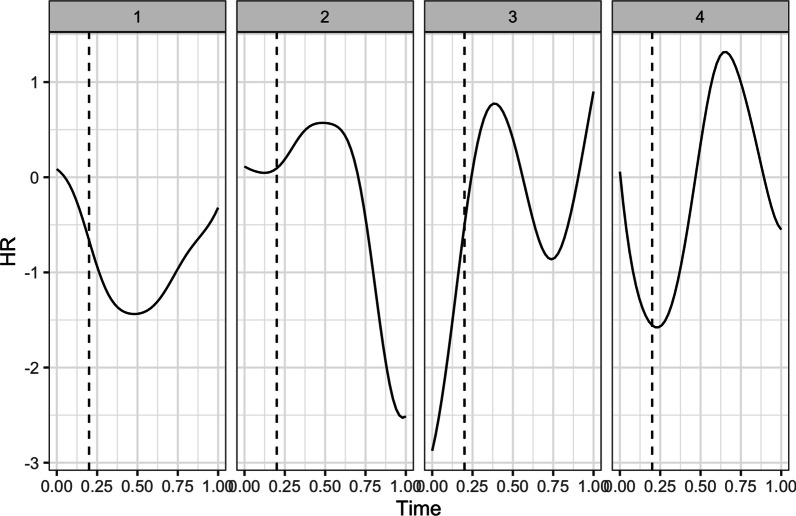
The four principal components that jointly describe the HR trajectories during and following the breach inducement. The vertical dashed line represents the end of the breach inducement

*Heart rate. *We performed two sets of fPCA: first to examine the effects of breach, zooming in on HR in the block prior to the manipulation and the block following the manipulation, and second to examine the effects of social accounts, focusing on the block prior to and following the social account manipulation. Starting with the effects of breach, the fPCA analysis showed that there were four principal components that together explained 94.53% of the variance in HR trajectories (see Fig. 3 for the raw HR trajectories). Figure 4 displays these four components. The first component explained 41.14% of the variance in HR trajectories and captures a decrease in HR during and after the breach inducement, followed by an increase towards the end of the subsequent block of trials. The second component explained 28.38% of the variance in HR trajectories and shows a small increase immediately following the breach inducement and a large decrease towards the end of the subsequent block of trials. The third component captures 14.86% of the variance in HR trajectories and illustrates a primary increase during the breach inducement, followed by a decrease, and finally a secondary increase towards the end of the block. The fourth and final component explains 10.14% of the variance in HR trajectories and captures a decrease during the breach inducement, an immediate increase afterwards, and again a decrease towards the end of the block.

Next, we ran a MANOVA with the four sets of principal component scores as the dependent variables, comparing the breach conditions to the fulfillment condition. Shapiro–Wilk tests showed that the residuals of the first (*W* = 0.99, *p* = 0.81), second (*W* = 0.98, *p* = 0.18), third (*W* = 0.99, *p* = 0.52), and fourth (*W* = 0.99, *p* = 0.95) principal component were normally distributed, whereas the Bartlett test showed that the homogeneity of variances assumption was met for the first (*K*^2^ = 1.56, *df* = 1, *p* = 0.21), second (*K*^2^ = 0.01, *df* = 1, *p* = 0.92), third (*K*^2^ = 3.39, *df* = 1, *p* = 0.07), and fourth (*K*^2^ = 0.61, *df* = 1, *p* = 0.44) principal component. With these assumptions met, we proceeded with the MANOVA, which confirmed that there was a significant difference in the dependent variables between the breach and the fulfillment conditions (*F*(4,100) = 3.09, *p* = 0.02, η^2^_G_ = 0.11). In particular, there was a significant difference between the breach and the fulfilment conditions on the first (*F*(1,103) = 6.30, *p* = 0.01) and the third (*F*(1,103) = 4.56, *p* = 0.04) principal component. The differences between the breach and fulfilment conditions were not significant for the second (*F*(1, 103) = 0.04, *p* = 0.85) and fourth (*F*(1, 103) = 0.86, *p* = 0.36) principal component.

**Fig. 5:**
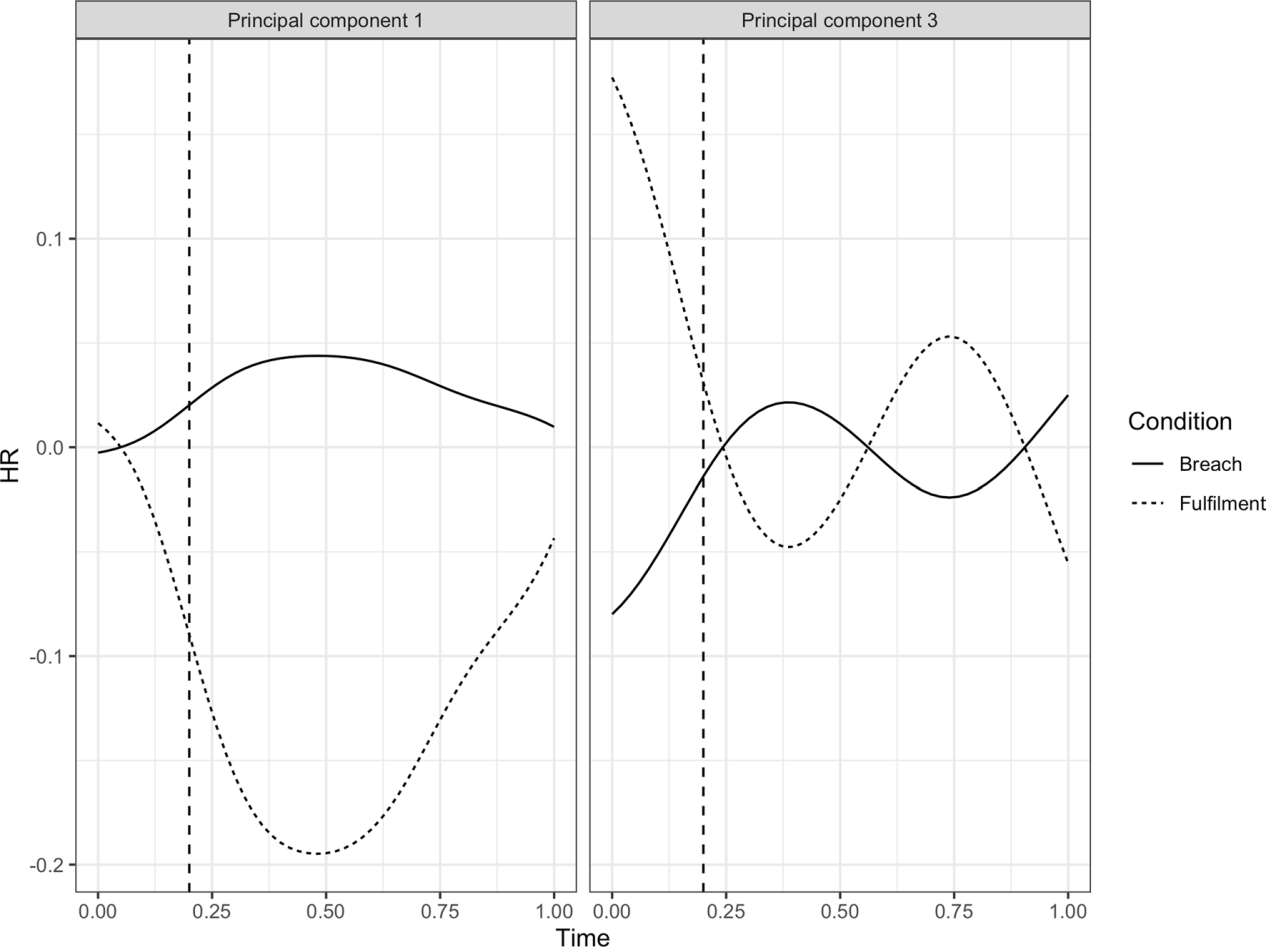
HR trajectories of first and third principal components for the breach and the fulfilment conditions

To interpret the significant differences, we plotted the HR trajectories of the first and third principal component for the breach and the fulfilment conditions (see Fig. 5). As can be seen in this figure, the fulfilment condition showed a substantial decline in HR on the first principal component, while the breach conditions experienced a minor increase in HR levels on this component. On the third principal component, the fulfilment condition showed an initial decrease, followed by a temporary increase in HR levels, whereas the breach conditions displayed an initial increase, followed by temporary decrease in HR levels. These results support our Hypothesis 1a stating that PCB cause elevated physiological levels, compared to PC fulfilment.

**Fig. 6:**
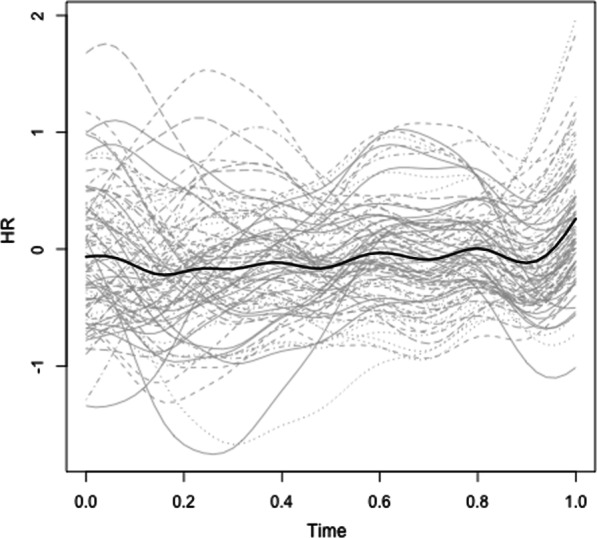
Individual (in grey) and average (in black) HR trajectories during and following the social account

**Fig. 7:**
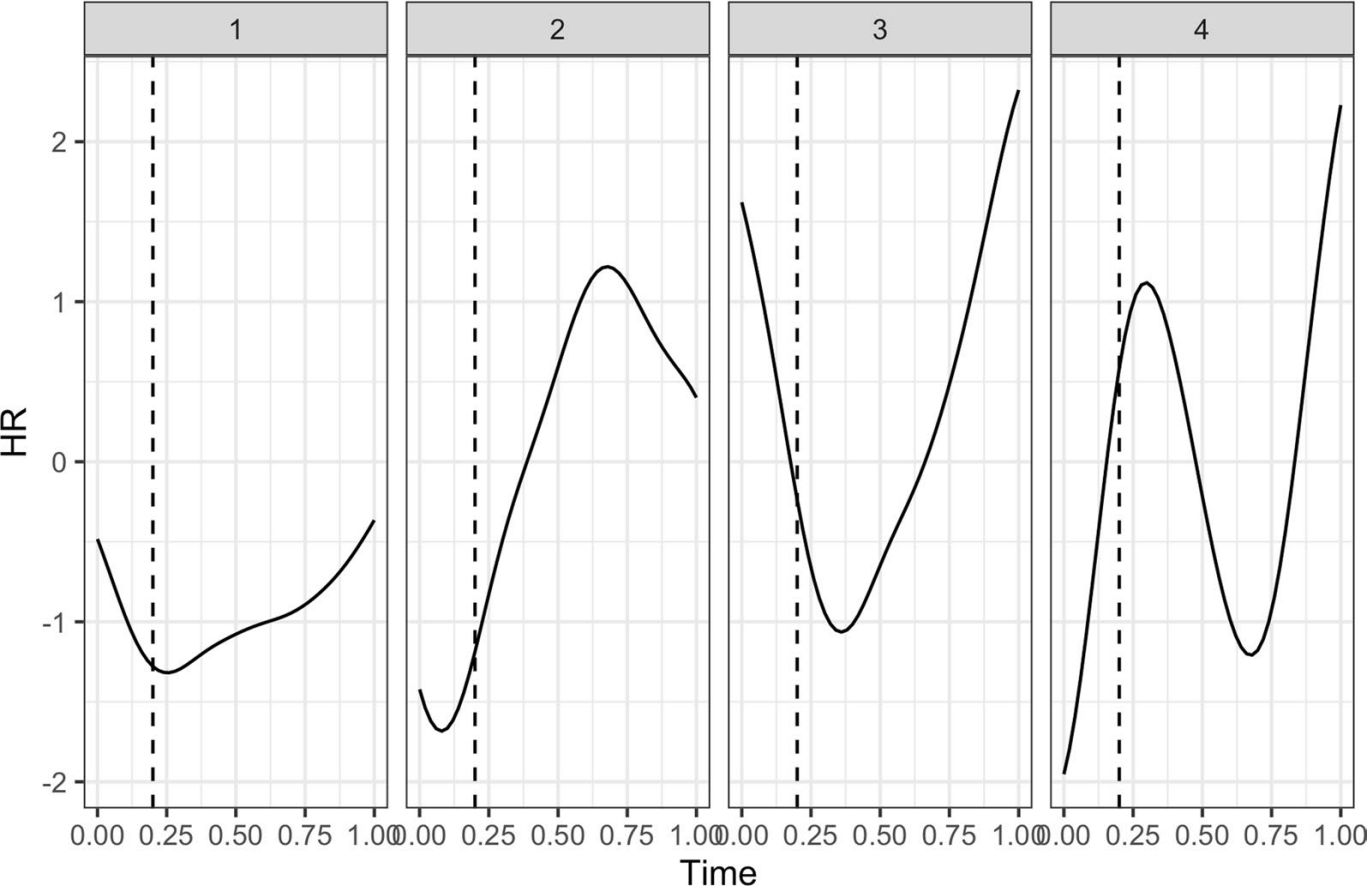
The four principal components that jointly describe the HR trajectories during and following the social account. The vertical dashed line represents the end of the social account manipulation

Next, we examined the effects of the social accounts on HR trajectories. For this analysis, we focused exclusively on the breach conditions, comparing the denial, apology, exonerating justification, blaming justification, and no social account conditions The fPCA again indicated that 4 principal components explained 93.19% of the variance in HR trajectories, thus exceeding the 90% threshold. Figure 6 shows the raw and average HR trajectories, whereas Fig. 7 displays the four principal components describing these HR trajectories. The first principal component (explained variance = 45.52%) shows a decrease in HR during the social account manipulation, followed by an increase towards the end of the block of trials. The second principal component (explained variance = 24.33%) captures an increase in HR starting during the social account manipulation and continuing afterwards, followed by a decrease midway the trials of the subsequent block. The third principal component (explained variance = 14.32%) represents a decrease in HR during the social account manipulation, followed by a steep increase in HR after the manipulation. The fourth and final principal component (explained variance = 9.03%) captures an increase in HR during the social account manipulation, followed by a decrease and a secondary increase midway the trials in the subsequent block.

Next, we ran a MANOVA with the four principal component scores as the dependent variables, comparing the denial, apology, exonerating justification, blaming justification, and no social account conditions to each other. Shapiro–Wilk tests showed that the residuals of the first (*W* = 0.99, *p* = 0.51), second (*W* = 0.99, *p* = 0.89), third (*W* = 0.99, *p* = 0.47), and fourth (*W* = 0.99, *p* = 0.71) principal component were normally distributed, whereas the Bartlett test showed that the homogeneity of variances assumption was met for the first (*K*^2^ = 1.38, *df* = 4, *p* = 0.85), second (*K*^2^ = 5.85, *df* = 4, *p* = 0.21), third (*K*^2^ = 2.14, *df* = 4, *p* = 0.71), and fourth (*K*^2^ = 4.30, *df* = 4, *p* = 0.37) principal component. With these assumptions met, we proceeded with the MANOVA which showed that there were no significant differences between the five conditions on these four principal components (*F*(4,81) = 1.40, *p* = 0.14, η^2^_G_ = 0.06). Further inspection of the principal components confirmed that there were no significant differences between the five conditions on the first (*F*(4,81) = 1.31, *p* = 0.27), second (*F*(4,81) = 1.56, *p* = 0.19), third (*F*(4,81) = 1.33, *p* = 0.27), and fourth (*F*(4,81) = 2.26, *p* = 0.07) principal component.

To better understand these null effects, we performed two additional exploratory MANOVAs. For the first exploratory MANOVA, we recoded social account conditions, creating a positive social account condition (i.e., apology and exonerating justification), a negative social account condition (i.e., denial and blaming justification), and a no social account condition, using these recoded conditions as independent variables. As assumptions of normally distributed residuals and homogeneity of variance were again met, we proceeded with a MANOVA. This MANOVA failed to reach statistical significance (*F*(2,83) = 1.43, *p* = 0.19, η^2^_G_ = 0.07), while further inspection confirmed that there were no significant differences between positive social accounts, negative social accounts, and no social accounts on the first (*F*(2,83) = 2.09, *p* = 0.13), second (*F*(2,83) = 2.66, *p* = 0.08), third (*F*(2,83) = 0.74, *p* = 0.48), and fourth (*F*(2,83) = 0.89, *p* = 0.41) principal component. For the second exploratory MANOVA, we again recoded the social account conditions, creating a condition in which a social account was offered (i.e., denial, apology, exonerating justification, blaming justification) versus a condition in which no social account was offered, using these recoded conditions as independent variables. As assumptions of normally distributed residuals and homogeneity of variance were again met, we proceeded with a MANOVA. This MANOVA failed to reach statistical significance (*F*(1,84) = 1.99, *p* = 0.10, η^2^_G_ = 0.09). Further inspection showed that there were no significant differences on the second (*F*(1,84) = 3.69, *p* = 0.06), third (*F*(1,84) = 0.54, *p* = 0.46), and fourth (*F*(1,84) = 0.18, *p* = 0.67) principal components. However, we did find a significant difference between the no social account and the social account conditions on the first principal component (*F*(1,84) = 4.20, *p* = 0.04).

**Fig. 8:**
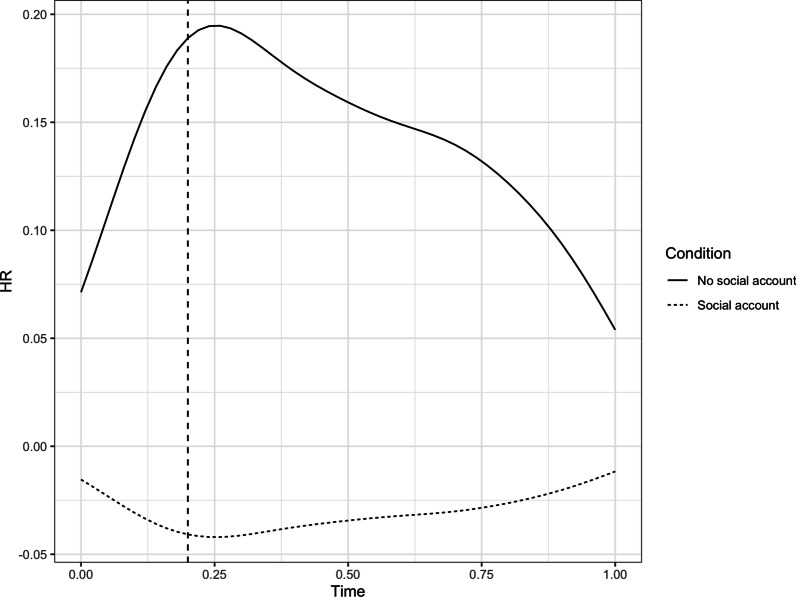
HR trajectories of the first principal component during and following social account for the social account versus no social account conditions. The vertical dashed line represents the end of the social account manipulation

To interpret this significant effect, we plotted the first principal component HR trajectory for the no social account and the social account conditions (see Fig. 8). As can be seen in this figure, the no social account condition displayed an increase in HR followed by a decrease once that participants continued with trials. In contrast, the social account conditions showed a small decrease in HR during the social account delivery, followed by a minor increase as participants moved on to complete further trials. While these exploratory results need to be interpreted with care, they tentatively suggest that offering no social account can trigger a brief increase in HR.

*Subjective stress *We first compared the impact of our breach manipulation on subjective stress by comparing the subjective stress score before and after the manipulation in the breach and the fulfilment conditions with a repeated-measures ANOVA. Results indicated that there were no main effects of condition (i.e., breach versus fulfilment; *F*(1, 102) = 2.01, *p* = 0.16, η^2^_*G*_ = 0.02) and time (i.e., before and after manipulation; *F*(1, 102) = 1.61, *p* = 0.21, η^2^_*G*_ < 0.001). There also was no significant interaction effect between condition and time (*F*(1, 102) = 0.00, *p* = 0.98, η^2^_*G*_ < 0.001). In summary, our manipulation of breach did not appear to alter participants’ subjective stress experiences, offering no support for Hypothesis 1b.

To assess the impact of social accounts on subjective stress, we performed a repeated-measures ANOVA, with time (comparing subjective stress scores given at the end of the block prior to the breach, the block following the breach and prior to the social account, and the block after the social account) as a within-subjects factor and condition (comparing the denial, apology, exonerating justification, blaming justification, and no social account conditions) as a between-subjects factor. Given that subjective appraisals of stress may be influenced by the sense-making process following breach perceptions, we added the scores on the breach measure as a covariate. Results showed that there were significant main effects of condition (*F*(4, 79) = 2.70, *p* = 0.04, η^2^_*G*_ = 0.11) and of time (*F*(1.78, 140.66) = 12.41, *p* < 0.001, η^2^_*G*_ = 0.02). The interaction between condition and time was not significant (*F*(7.12, 140.66) = 1.71, *p* = 0.11, η^2^_*G*_ = 0.01). To interpret the significant main effect of condition, we performed ten pairwise post-hoc tests with Holm correction. Results showed that subjective stress was, on average, significantly higher in the denial condition compared to the exonerating justification condition (*M*_*denial*_ = 5.63, *M*_*exonerating justification*_ = 3.81, *t*(79) = 3.05, *p* = 0.03). None of the other comparisons reached statistical significance. To interpret the significant main effect of time, we performed three pairwise post-hoc tests with Holm correction. Results showed that subjective stress was, on average, significantly lower after the breach induction and prior to the social account (*M* = 4.56) compared to prior to the breach (*M* = 5.05, *t*(158) = 3.54, *p* = 0.001) and it was also significantly lower after the social account (*M* = 4.39) compared to prior to the breach (*M* = 5.05, *t*(158) = 4.81, *p* < 0.001). There was no significant difference when comparing stress levels just before the social account to after the social account (*t*(158) = 1.26, *p* = 0.21). Overall**,** the non-significant interaction effect suggests that we cannot support Hypotheses 2–5.

## Discussion

In the present study we aimed to investigate how physiological (i.e., HR) and psychological or subjective (i.e., self-reported) stress reactions following PCB unfold over time. Moreover, we analyzed how these changes in reactions can be explained by means of providing social accounts (i.e., denial, exonerating justification, blaming justification, apology, and no social account).

Since subjective stress was assessed directly after the PCB/social account feedback, it can be considered as a first phase of the sense-making process participants will go through. Based on these ratings, we were not able to find support for our hypotheses. More specifically, participants did not report higher subjective stress levels when experiencing PCB compared to PC fulfillment (Hypothesis 1b) and the social accounts presented in this study did not cause significant stress increases (Hypothesis 2 and 4) or decreases (Hypothesis 3 and 5) compared to the control condition. However, as mentioned earlier, subjective stress ratings may be subject to systematic biases [41]. For example, it can be argued that individuals might use emotional-suppressing strategies in order to reduce emotional expressiveness when it comes to negative emotions and stress. Due to their social undesirability, negative emotions and stress are more prone to be suppressed in view of protecting one’s self-image and avoiding vulnerable emotional states. Therefore, it might occur that a person objectively displays a strong physiological arousal without necessarily reporting their somatic changes as “stress” [9]. For instance, based on participants’ HR, we did find that PCB caused an elevated physiological reaction compared to PC fulfillment (Hypothesis 1a). However, our HR-findings could not provide support for which social accounts may aggravate or, in contrast, aid individuals stress recovery. As a matter of fact, it seems that after a short-lasting stress reaction, participants’ HR decreased over the course of the experiment. These findings can be understood along previous research [3, 20, 42] demonstrating that after an acute increase in HR as a reaction to a psychological stressor, individuals’ HR showed a decline over time, even when the subjects were exposed to prolonged stress. These results showing an adaptation to stress are considered as normal since without physiological compensation there could be escalating, detrimental effects on the organism [3]. Based on this, it seems that the use of an alternative physiological indicator of stress might offer a more reliable indication of how social accounts can affect one’s stress recovery. For example, subject’s blood pressure (BP was shown to offer a more accurate picture of the stress recovery process (i.e., increase in PB indicates aggravation, while decrease in BP indicates recovery, [42]. Similarly, heart rate variability (HRV) measures seem more suitable to detect fluctuations in stress in reaction to recovery efforts such as social accounts [36]

Further, it can be argued that one’s interpretation of the incoming information is also relevant in examining reactions to social accounts. More specifically, the causal explanation individuals give to interpret the reasons for PCB will also influence their subsequent reactions [64]. Previous research has demonstrated that social accounts are not always received or remembered as meant [54] For example, it is possible that the blaming justification or the apology offered by the experimental leader had no significant effect on the participants’ stress resolution because they were not interpreted as intended. In line with trust repair literature, we propose that an attributional perspective could offer further insights into the examined stress resolution processes (e.g., [34]. According to Weiner’s [71] causal attribution theory, individuals interpret the social account based on three primary, continuous and interdependent attribution dimensions: locus of causality, controllability, and stability [71]. *Locus of causality* refers to whether the blame for the PCB is located internally (i.e., the organization) or externally (e.g., economic climate). *Controllability* indicates whether the PCB can be assigned to factors controlled by the organization or by another party. *Stability* refers to the extent to which the reason underlying the PCB will either fluctuate or remain stable over time. Turning to our experiment, it can be argued that our participants after hearing one of the proposed social accounts, assigned their own meaning to the incoming information about the cause behind the PCB. For example, even though the experimental leader intended to exonerate himself of the blame when presenting an exonerating social account, following Weiner’s causal attribution theory, it is the participants’ interpretation that will determine the effects of the social account on the stress resolution process. Several PC scholars already examined the role of attributions in the aftermath of PCB (e.g., [12, 68]. However, a further integration of social accounts, causal attribution and stress is needed to better understand the underlying mechanisms of the stress resolution process following organization’s attempts to account for PCB.

## Theoretical implications

We contributed to the literature by adopting a process-oriented approach to explore within-person changes in stress experiences. In doing so, we respond to recent calls for more time-sensitive and dynamic research in the PC literature [1, 53, 57, 66]. Moreover, our study further extends the Post-Violation Model [66]. This model provides a theoretical framework of distinct ways in which employees may react to PCB by engaging in a self-regulation process resulting in a range of plausible outcomes. As such, some individuals may end up with a similar PC (i.e., reactivation) as before the PCB, while others experience a more beneficial (i.e., thriving) or a less favorable (i.e., impairment) PC that the original one. In some cases, employees are incapable of forming a functional PC resulting in experiences of behavioral and/or mental disengagement (i.e., dissolution). While the Post-Violation Model focuses primarily on the end state of psychological contracts, our research goes a step further and examines the recovery process stretching out over a period of time (i.e., immediate and delayed secondary responses, [28]. Differentiating these reactions is important since prolonged stress reactions impacts individual’s wellbeing [46].

## Limitations and future directions

Our study was conducted in a laboratory setting in which students were engaged in a short-term employment relationship. Yet, psychological contract theory does not exclude students or short-term work. As such, the cognitive and/or physiological processes should take place regardless of the student status or the duration of the employment relationship. Moreover, the experimental setting adopted for this study allowed us to control for possible confounders and to manipulate the key features central to an employment relationship [40]. Nevertheless, manipulating PCB in a lab setting introduces certain issues. For example, the breach event that was created in our experiment may not have the same impact as a major real-life breach event, such as not getting a promotion. Hence, this may have led to weaker effects of the breach manipulation on heart rate and subjective stress. We therefore encourage future research to replicate these findings in field settings, that is, examining full-time employees in organizations, as they offer a broader repertoire of different PCB types and alternative recovery efforts.

Next, this study exclusively focused on a *transactional* exchange, even though many employment relationships entail more than this exchange type only. Hence, our experiment could not capture the wealth of exchanges that exist between an organization and its employees. Indeed, most employment relationships feature a more complex *relational* exchange, in which socioemotional incentives such as emotional support and job security are offered in exchange for contributions such as commitment and loyalty. Future experimental and field research should therefore study more elaborated and realistic exchanges yielding more than one contribution or inducement. Indeed, an employment relationship can hardly be scaled down to a single exchange, but rather a series of exchanges from both parties. Moreover, both prior and future exchange experiences might also affect employees’ reactions. For instance, an employee who has continuously received high levels of inducements will react differently compared to an employee who is used to receive lower inducement levels. Such consequences can be examined in organizational settings, using repetitive measures over a period of time [40].

Further, following the PVM, it is likely that the stress resolution process will not only be influenced by the type of organizational response but also by the relative speed of resolution efforts (i.e., resolution velocity; [66]. *Resolution velocity* refers to the relative speed of resolution as perceived by the victim during the post-breach recovery process. For instance, an employee might believe that a stressful encounter such as PCB can be successfully resolved if redress occurs fast enough, compared to his/her standards [21, 65]. Such time-related effects are scarce in PC literature and should therefore be examined in future research. This knowledge can help to gain more insight in the stress resolution process and explain why some employees might recover faster, while others end up in a state of chronic stress.

Moreover, the current research can be considered as an important first step towards a process-oriented approach to explore within-person changes in stress experiences. However, given the relatively small sample size, our findings should be interpreted with caution until replicated with more robust methodologies and greater statistical power. The relatively low power of our analyses may have led to type II errors, which may explain some of the null effects. We had sufficient power to detect medium-to-large effects, but it is possible that our experimental design only elicited small effects which we may not have been able to pick up. Future studies could use larger samples in lab-based experiments or could make use of large-scale surveys among different organizations and/or daily diary studies assessing unfolding stress reactions following PCB and organizational social accounts.

Also, our findings, in line with previous research demonstrated that participants’ HR displayed a natural decline pattern over time as a result of adaptation to stress. We encourage future studies to use more sensitive physiological indicators of stress such as BP and HRV to examine how social accounts might affect physiological stress recovery processes in the aftermath of PCB.

Finally, the unfolding nature of physiological and psychological stress reactions to PCB was measured through a short time interval during the course of the experiment. In a real work setting these unfolding reactions might last days, weeks, or even months. We therefore recommend future research to replicate our study in field settings that capture more realistic timespans following PCB perceptions.

### Practical implications

Based on our results, PCB will trigger a short-lasting increase in HR.

Even though these responses were of relatively short duration, these repeated fluctuations could have pathological consequences [3]. Research has shown that the prevalence of PCB is quite high, with employees perceiving PCB at least once a week [14]. Organizations should therefore be aware of the negative consequences of these repeated perceptions of PCB

Furthermore, it can be argued that the individual plays an active role in effective stress management [35, 44]. Therefore, we encourage organizations to also train employees to be aware of these reactions and cope adequately when perceiving PCB. For example, employees can be trained to use efficient coping mechanisms such as problem-focused approaches or to apply adequate cognitive reappraisal strategies that may protect them against stress [10, 23].

## Conclusions

The current research allows us to demonstrate that PCB will trigger a short-lived increase in heart rate. Further research is needed to better understand unfolding trajectories of physiological reactions to contract breach and the effect of social accounts as organizational recovery efforts.

## Data Availability

The datafiles and the R scripts used to analyze the data can be downloaded from https://osf.io/qj86m/?view_only=8af8ef16ee3340a48b1ccb217bafd5e2. Descriptive statistics and correlations between key variables can be consulted in the Supporting information.

## Appendix 1

### Psychological contract breach measure

Please indicate the extent to which you agree with following statements

**Table:**

1	2	3	4	5
Totally disagree	Somewhat disagree	Nor disagree nor agree	Somewhat agree	Totally agree

**Table:**

	1	2	3	4	5
Overall, the experimental leader fulfilled her commitments to me	□	□	□	□	□
In general, the experimental leader lived up to her promises to me	□	□	□	□	□

### Subjective stress measure

Please indicate your current level of stress

**Table:**

Totally not stressed								Extremely stressed
1	2	3	4	5	6	7	8	9

### Feelings of violation measure

Please indicate the extent to which you agree with following statements

**Table:**

1	2	3	4	5
Totally disagree	Somewhat disagree	Nor disagree nor agree	Somewhat agree	Totally agree

**Table:**

	1	2	3	4	5
I feel a great deal of anger towards the experimental leader	□	□	□	□	□
I feel betrayed by the experimental leader	□	□	□	□	□
I have the feeling that the experimental leader has violated the contract between us	□	□	□	□	□
I feel extremely frustrated with how I was treated by the experimental leader	□	□	□	□	□
The experimental leader broke the promise made with regards to the promised compensation	□	□	□	□	□

## Appendix 2

Exact wording of different social accounts:

- **Denial**: “You indicated that the experimenter did not fulfill all of her obligations towards you. I don’t know which unfulfilled obligations you are referring to”
  - **Apology**: “You indicated that the experimenter did not fulfill all of her obligations towards you, I am truly sorry to hear that you have to go through this disagreement”
    - **Blaming justification**: “You indicated that the experimenter did not fulfill all of her obligations towards you, The reason for this is that I can’t afford to pay you more money, since I invited too many participants today and I haven’t foreseen enough money to pay them all”
      - **Exonerating justification**: “You indicated that the experimenter did not fulfill all of her obligations towards you. The reason for this is that I can’t afford to pay you more money as I just saw that my lab-mate used my money to pay his own participants”

## Abbreviations

ANS: Autonomous nervous system
BP: Blood pressure
PC: Psychological contract
PCB: Psychological contract breach
COR: Conservation of resources
PVM: Post-violation model
ECHW: Ethische Commissie Humane Wetenschappen
HR: Heart rate
HRV: Heart rate variability
FDA: Functional data analysis
fPCA: Functional principal component analysis
ANOVA: Analysis of variance
MANOVA: Multivariate analysis of variance
